# Encapsulation of Curcumin in Polystyrene-Based Nanoparticles—Drug Loading Capacity and Cytotoxicity

**DOI:** 10.1021/acsomega.1c00867

**Published:** 2021-04-29

**Authors:** Maria Zatorska-Płachta, Grzegorz Łazarski, Urszula Maziarz, Aleksander Foryś, Barbara Trzebicka, Dawid Wnuk, Karolina Chołuj, Anna Karewicz, Marta Michalik, Dorota Jamróz, Mariusz Kepczynski

**Affiliations:** † Faculty of Chemistry, Jagiellonian University, Gronostajowa 2, Kraków 30-387, Poland; ‡ Centre of Polymer and Carbon Materials, Polish Academy of Sciences, M. Curie-Sklodowskiej 34, Zabrze 41-819, Poland; § Department of Cell Biology, Faculty of Biochemistry, Biophysics and Biotechnology, Jagiellonian University, Gronostajowa 7, Kraków 30-387, Poland

## Abstract

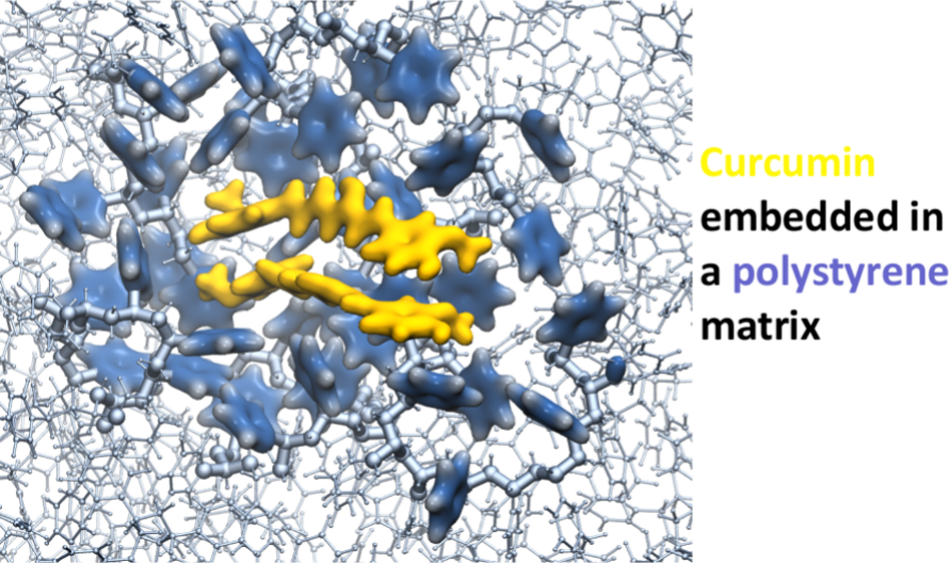

Nanoparticles made of amphiphilic block copolymers are commonly used in the preparation of nano-sized drug delivery systems. Poly(styrene)–*block***–**poly(acrylic acid) (PS–PAA) copolymers have been proposed for drug delivery purposes; however, the drug loading capacity and cytotoxicity of PS–PAA nanoparticles are still not fully recognized. Herein, we investigated the accumulation of a model hydrophobic drug, curcumin, and its spatial distribution inside the PS–PAA nanoparticles. Experimental methods and atomistic molecular dynamics simulations were used to understand the molecular structure of the PS core and how curcumin molecules interact and organize within the PS matrix. The hydrophobic core of the PS–PAA nanoparticles consists of adhering individually coiled polymeric chains and is compact enough to prevent post-incorporation of curcumin. However, the drug has a good affinity for the PS matrix and can be efficiently enclosed in the PS–PAA nanoparticles at the formation stage. At low concentrations, curcumin is evenly distributed in the PS core, while its aggregates were observed above ca. 2 wt %. The nanoparticles were found to have relatively low cytotoxicity to human skin fibroblasts, and the presence of curcumin further increased their biocompatibility. Our work provides a detailed description of the interactions between a hydrophobic drug and PS–PAA nanoparticles and information on the biocompatibility of these anionic nanostructures which may be relevant to the development of amphiphilic copolymer-based drug delivery systems.

## Introduction

Polymeric nanoparticles (NPs) have attracted a great deal of attention as nanocarriers of drugs or biomolecules (e.g., proteins and peptides). Amphiphilic block copolymers (AmBCs, containing both the hydrophobic and hydrophilic blocks) have been used to prepare a range of different NPs.^1^ It has been shown that, depending on factors such as mass fraction of the hydrophilic block (*w*_hydrophilic_) and a method of preparation, AmBCs can self-organize in an aqueous environment to form micelles, rods, core–shell NPs, nanocapsules, and vesicles (polymersomes).^2^ All these structures consist of a hydrophobic core capable of encapsulating hydrophobic bioactive substances (e.g., drugs) and a hydrophilic corona ensuring the stability of NPs in water.^3^ The ability of NPs to accumulate a drug is quantified by the drug-loading capacity (DLC, defined as the ratio of the weight of the drug entrapped in the polymer phase to the weight of the polymer). Factors that affect DLC include the chemical nature of the solute and the core-forming block, the molecular weight of the copolymer, and, to a lesser extent, the nature and length of the corona-forming block.^4,5^ However, the most important factor is the compatibility of the drug with the core-forming block.^6^ Understanding of the polymer–drug interactions and the molecular structure of the hydrophobic core is, therefore, a very important factor in the development of effective NP-based drug carriers.

Poly(styrene)–*block***–**poly(acrylic acid) (PS–PAA) is one of the most frequently studied AmBCs.^7^ Although PS–PAA copolymers have been proposed for drug delivery purposes before,^8^ there are several examples of their application in this field described in the literature so far. Micelle-like PS–PAA NPs have been recently investigated as possible carriers of clonazepam,^9^ a category D antiepileptic agent. The authors showed that the DLC of the PS–PAA NPs for this drug is very low (less than 1%). It was suggested that clonazepam localizes between the PAA chains in the corona, rather than in the core, as a result of hydrogen bonding and electrostatic interactions between the PAA carboxyl functional groups and the drug. In another study, Caon et al. used PS–PAA polymersomes as an effective carrier of finasteride (FIN), a drug used to treat prostatic hyperplasia and androgenetic alopecia.^10^ The chitosan-coated FIN-containing polymersomes were shown to be very well suited for topical drug administration since such a formulation allows for a controlled release and enhanced retention of FIN in the dermis and epidermis. In addition, a recent study has shown that PS–PAA NPs are a promising nanostructured carrier of hydrophobic photosensitizers used in photodynamic therapy (PDT).^11^ The authors showed that PS-*b*-PAA NPs are not only biocompatible but also have the ability to permeate the superficial layer of the dermis and therefore are suitable for both topical and intravenous administration of PDT photosensitizers. All these studies demonstrate a significant potential of using PS–PAA NPs in drug delivery and the need for in-depth research of the interactions between PS–PAA and hydrophobic biologically active agents.

What is more, polystyrene nano- and microspheres have previously been investigated as carriers for drugs such as ibuprofen,^12^ indomethacin,^13^ and progesterone.^14^ Amino or carboxyl modified polystyrene structures have also been applied to deliver chondroitin sulfate A,^15^ antigens, peptides, and proteins.^16^ In turn, PAA was applied as a hydrophilic block in other AmBCs used as the model drug nanocarriers.^17,18^ Crosslinked pluronic-*g*-PAA microparticles (MPs),^19^ as well as PAA-functionalized Co_0.85_Se NPs,^20^ have recently been used successfully as pH-sensitive drug delivery systems for doxorubicin. Carbopol polymers, which are high molecular weight crosslinked homopolymers and copolymers of PAA, have also been used as drug delivery systems^21,22^ and have been shown to enhance mucoadhesion and cell internalization of the NPs.^23^

In this work, we used both experimental methods and computer simulations to study the molecular structure of the hydrophobic core of the PS–PAA NPs and the interaction of a hydrophobic drug with this core. Curcumin (Cur) was used as a model drug. Cur is a natural polyphenol known for its anti-cancer, anti-inflammatory, and antioxidant properties.^24^ Due to its low solubility in an aqueous environment, as well as its low stability in neutral and alkaline solutions, Cur requires an appropriate carrier to be effectively delivered to the human body. We mainly considered the effect of Cur entrapment method on drug accumulation and its spatial distribution in the polymer matrix. The cytotoxicity of the empty and Cur-containing nanospheres obtained from PS–PAA was also studied. Overall, our work provides a detailed description of the interactions between the hydrophobic drug and PS–PAA NPs and information on the biocompatibility of these anionic nanostructures.

## Molecular Dynamics Simulations

### Models and Parameterization

The parameterization of curcumin and its anion in the all-atom CHARMM36 force field^25^ was described previously.^26^ The Cur molecule was assumed to be in its enolic tautomeric form according to the experimental findings.^27^ In addition to the neutral curcumin (Cur^0^), its anionic form (Cur^–^) was taken into consideration because the p*K*_a_ value for the enol hydrogen (8.38 ± 0.04^28^) indicates a non-negligible concentration of the deprotonated form at the physiological pH (ca. 9% of the monoanionic form resulting from dissociation of the enolic OH function^29^).

Polystyrene was modeled by 40 unit oligomers. To reflect the atacticity of the polymer chain, two PS units with different absolute configurations around the central carbon atom were generated (STYRA and STYRB, see the Supporting Information) and joined in a random manner on keeping the approximate 1:1 ratio of both units along the chain. The structure of the units reflects the real atactic polymers as they were linked in a head-to-tail manner with an approximately 1:1 ratio of different aromatic ring configurations. Seven such oligomers with various (*R*,*S*) sequences along the chain were produced. The oligomers were parameterized with the all-atom CHARMM36 force field.^25^ The types of atoms used are shown in Figure S1 and Table S1. Each of the seven oligomers was simulated under vacuum for 10 ns. Two structures were extracted from each trajectory, giving 14 different initial oligomers configurations. Water was described with the CHARMM-specific TIP(S)3P model. Na^+^ cations were described by the CHARMM36 parameters.

### System Preparation

The simulated systems are summarized in Table 1. To obtain the PS aggregate, the above-mentioned 14 PS oligomers were placed randomly in a simulation box, which was filled with water (approx. 105 000 molecules) yielding the PS_coil system (Figure S2). The system was optimized to minimize its energy and then simulated for 200 ns. The resulting polymer aggregate was used as part of some of the Cur-containing systems.

**Table 1:** Summary of the Simulated Systems^a^

system	PS form	Cur form	solvent	simulation length (ns)
PS_coil	14 dispersed oligomers		∼105 000 H_2_O	200
Cur^0^–PS_coil	cluster of 14 oligomers	12 Cur^0^	∼105 000 H_2_O	3 × 200
Cur^0^–PS_disp	14 dispersed oligomers	12 Cur^0^	∼105 000 H_2_O	3 × 500
Cur^–^–PS_coil	cluster of 14 oligomers	12 Cur^–^	∼105 000 H_2_O + 12 Na^+^	3 × 200
Cur^–^–PS_disp	14 dispersed oligomers	12 Cur^–^	∼105 000 H_2_O + 12 Na^+^	3 × 200

a The table shows the number of molecules in the given system and the simulated time scale.

**Figure 1:**
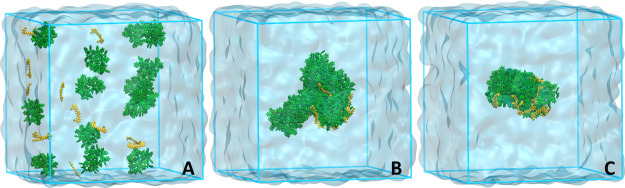
Snapshots of the configurations of one of the Cur^0^–PS_disp systems at *t* = 0 (A), 200 ns (B), and 500 ns (C). The Cur molecules and PS oligomers are shown in yellow and green, respectively.

**Figure 2:**
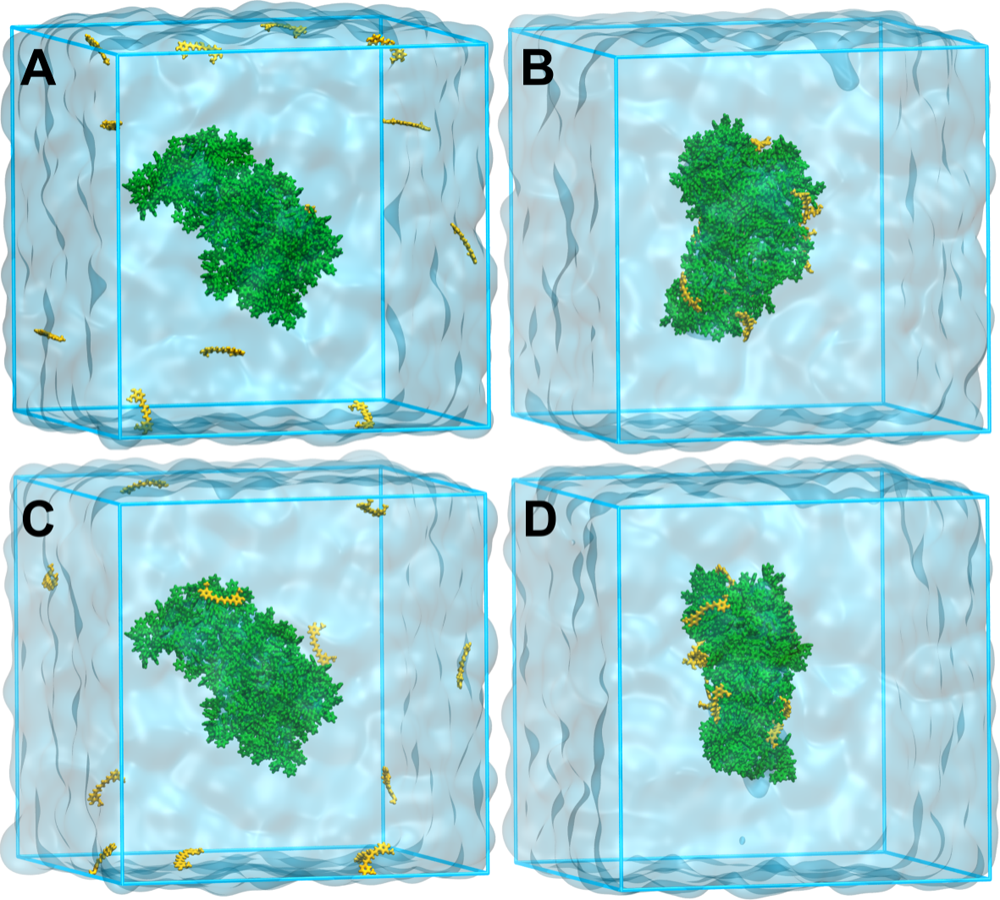
Snapshots of the configurations of systems Cur^0^–PS_coil (A,B) and Cur^–^–PS_coil (C,D) at *t* = 0 and 200 ns. The Cur molecules and PS oligomers are shown in yellow and green, respectively.

Cur-containing systems were constructed in two ways: (1) 12 Cur molecules were placed between 14 PS oligomers in a simulation box (which corresponds to ∼7.6 wt % of Cur) prior to their aggregation (the PS_disp systems, Figure 1A) and (2) 12 Cur molecules were added to the simulation box with the already formed aggregate (the PS_coil systems), as shown in Figure 2A. Each system was represented by three different initial positions of the molecules and two Cur ionization states, giving 12 systems in total (Table 1).

### Simulation Details

All the generated systems were optimized to minimize their energy and then subjected to a preliminary 10 ns *NpT* simulation. The temperature (298 K) and the pressure (1 bar) were controlled by the Berendsen thermostat and barostat, respectively. Production simulations were carried out for 200 ns (extended by 300 ns in some cases) with the same intensive parameter values controlled by the velocity rescale thermostat^30^ and the Parrinello–Rahman barostat.^31^ The van der Waals potential was calculated with a cutoff at 1.2 nm modified by the force-switch function with a switch distance of 1.0 nm. The electrostatic interactions were computed using the particle mesh Ewald (PME) algorithm with the Coulomb cutoff radius of 1.0 nm. The hydrogen bond lengths were subjected to constraints by the LINCS algorithm,^32^ allowing for a 2 fs time step. The simulations and most of the analysis were performed using the GROMACS 2018 software package.^33,34^ The “free volume” analysis tool provided in the GROMACS package was used in density calculations. Trajectory visualizations were made using the VMD package.^35^ Density maps were prepared using a “volmap” tool available as a part of the VMD package to generate volumetric data and subsequently visualized using a custom program.

## Results and Discussion

### Self-Assembly of PS–PAA in Aqueous Media

**Figure 3:**
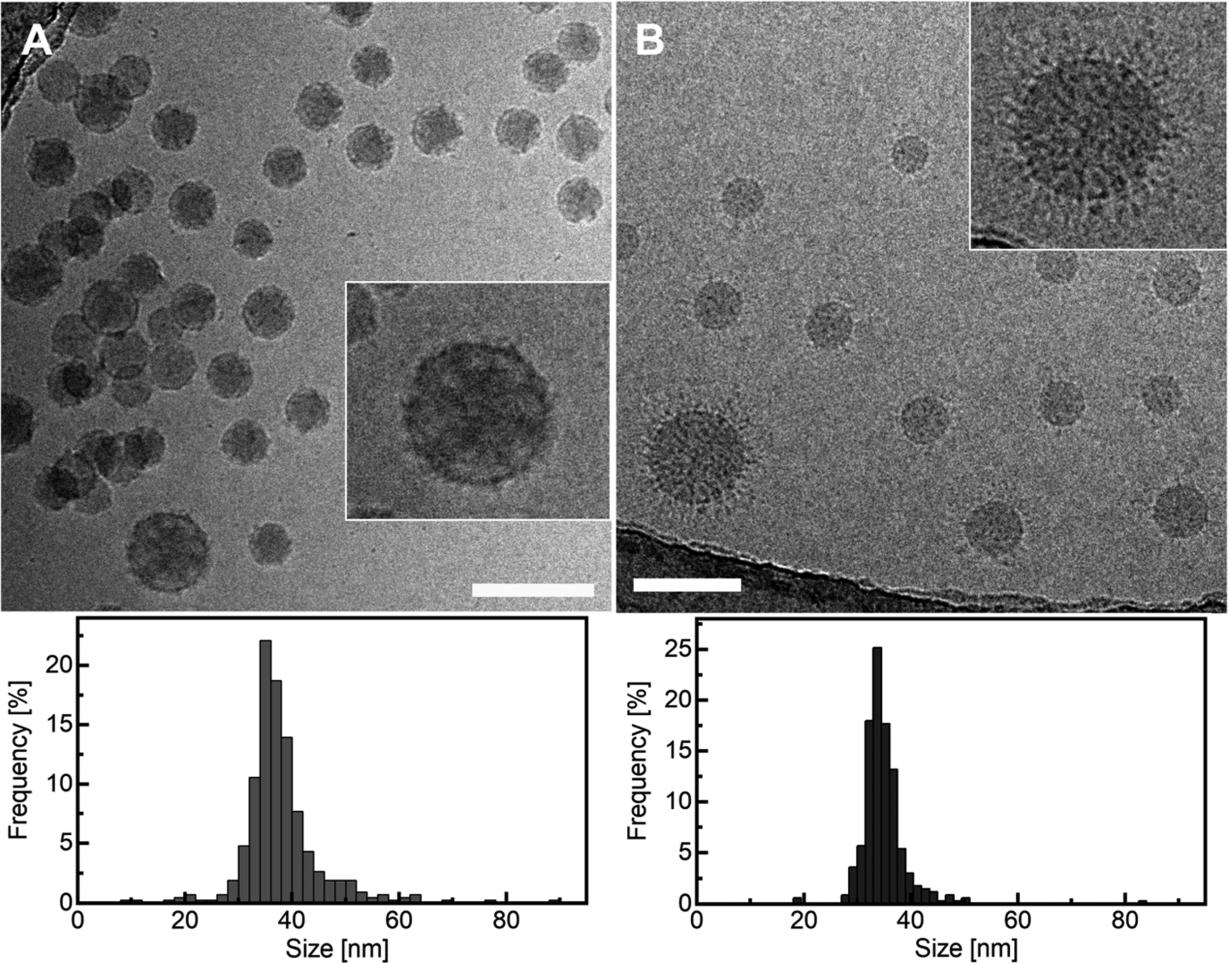
Cryo-TEM micrographs and corresponding diameter distributions of the PS–PAA NPs (*c*_PS–PAA_ = 0.7 mg/mL) at pHs 4.1 (A) and 6.5 (B). Scale bars correspond to 100 nm.

Depending on the block length and preparation method, PS–PAA copolymers can self-assemble into various nanostructures in an aqueous environment.^7^ Therefore, we first examined the morphology of the objects formed in the PS–PAA dispersion using cryo-TEM microscopy. Figure 3 shows typical aggregates formed in the PS–PAA dispersion (*c*_PS–PAA_ = 0.7 mg/mL) obtained by dialysis at pH 4.1 and 6.5. Spherical particles ranging in size from 20 to 60 nm (averaged diameter = 38 ± 7 nm) are present in the dispersion at pH 4.1. Some NPs stick to each other indicating a tendency to aggregate. The enlargement of one of the NPs (Figure 3A) shows that the NPs have an internal structure with lighter areas that are likely internal reverse micelles formed by the PAA blocks and filled with water and darker ones corresponding to the polystyrene shells. The surface of the spheres is covered with a coating composed of the hydrophilic PAA blocks, which makes them stable in an aqueous solution. A similar internal structure was previously described for another PS–PAA copolymer.^7^ At the higher pH, the NPs are well separated, while the core sizes are similar to those at low pH (averaged diameter = 35 ± 5 nm). In addition, a polymeric corona is visible on their surface. The PAA block, as a weak polyelectrolyte, is susceptible to dissociation, and its conformation should be strongly pH dependent. The p*K*_a_ value for PAA is ca. 4.5;^36^ thus, at lower pH values, the carboxyl groups of the PAA chains are predominantly undissociated, and the hydrophilic blocks adopt tightly coiled conformations. With increasing pH, the carboxyl groups dissociate, and at pH 6, the PAA blocks are almost completely ionized. The chains expand due to the repulsion of charged species along the chain, which was observed as the corona around the NPs.

It is known that the balance of hydrophilic/hydrophobic segments (referred to as the mass fraction of the hydrophilic block, *w*_hydrophilic_) has a significant impact on the morphology of the objects observed in copolymer dispersions.^2^ Spherical and worm-like micelles, vesicles, and solid-like particles can be formed depending on the *w*_hydrophilic_ value. In our case, the corona-forming block (PAA) is short compared to the core-forming block (PS), and *w*_hydrophilic_ is ca. 0.15. There are several studies in the literature on the PS–PAA morphologies in dispersions; however, most of them were performed in organic solvent/water mixtures. Vilsinski et al. showed, using conventional TEM microscopy, that the PS–PAA 70.500:13.000 copolymer can form monodisperse micelle-like nanostructures with an average size of less than 50 nm,^11^ which is consistent with our observations. Self-aggregation of PS–PAA copolymers into micelles with the formation of the PS core and the PAA corona was also demonstrated using molecular dynamics (MD) simulations,^37^ which showed that the corona-forming PAA blocks in the protonated state adopt coiled conformations while in the fully dissociated states have extended conformations.

### Incorporation of Cur into PS–PAA NPs

To test the ability of the PS–PAA NPs to accumulate drugs, we encapsulated Cur in their hydrophobic core. The hydrophobic compound can be incorporated into the hydrophobic core of polymer nanostructures either during their formation or later, as a result of its interaction with the already formed nanostructures. We verified both approaches. The PS–PAA NPs loaded with Cur were prepared by dialysis of a solution containing the copolymer and the drug in polar organic solvents against water. A series of samples with a constant PS–PAA concentration and a Cur content of 8–30 wt % relative to the polymer weight were prepared. UV–vis measurements were used to evaluate the DLC and EE values for Cur encapsulated in the PS–PAA NPs (Table 2). The DLC values show that the PS–PAA NPs can accumulate up to 11 wt % of the drug with respect to the copolymer weight during the NP preparation.

**Table 2:** DLC and EE of Curcumin in PS–PAA NPs and PS MPs

sample	Cur load^a^ [wt %]	DLC [%]	EE [%]
PS–PAA NPs	8	4.4 ± 0.7	40.9 ± 13.1
PS–PAA NPs	30	11.5 ± 1.8	25.7 ± 2.2
PS MPs	5	1.6 ± 0.2	23.1 ± 10.4
PS MPs	10	5.4 ± 1.0	51 ± 13.6
PS MPs	30	24.2 ± 3.2	76.4 ± 13.5

a Weight ratio of Cur and PS–PAA or PS in the feed.

**Figure 4:**
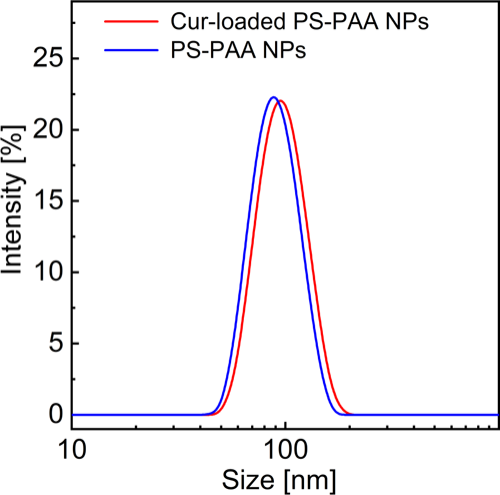
Hydrodynamic diameter distributions for PS–PAA NPs (*c*_PS–PAA_ = 0.22 mg/mL) and curcumin-loaded PS–PAA NPs (with about 8 wt % of Cur) measured by DLS experiments at pH 7.4.

The average hydrodynamic diameter (*d*_z_) measured by the dynamic light scattering (DLS) technique for the PS–PAA NPs and Cur-loaded NPs was 87.2 ± 1.3 and 93.6 ± 1.7 nm, respectively. Figure 4 shows the distribution profiles of *d*_z_. The DLS results indicate that the incorporation of Cur during the preparation of the PS–PAA NPs had only a little effect on the particle size.

We also checked the possibility of Cur encapsulation into PS–PAA NPs already existing in the dispersion. The empty NPs were incubated for 2 h in a Cur solution, and the drug fluorescence spectrum was measured (Figure S3). Although it is well established that the intensity of Cur fluorescence increases significantly upon partitioning into a less polar environment,^38,39^ we observed only slight changes in the Cur emission spectrum, even when a high concentration of the PS–PAA NPs was added to its aqueous solution. This clearly shows that Cur did not interact with the PAA corona and it cannot be encapsulated in the PS–PAA NPs after their formation. One possible explanation is that the PS cores are below their glass-transition temperature (*T*_g_), and their structure is very rigid. The Cur molecules incorporated in the PS–PAA NPs during their formation change the structure of the PS core, which facilitates drug accumulation. These changes are impossible after the NP formation; therefore, the penetration of the interior of PS–PAA NPs by Cur is difficult.

**Figure 5:**
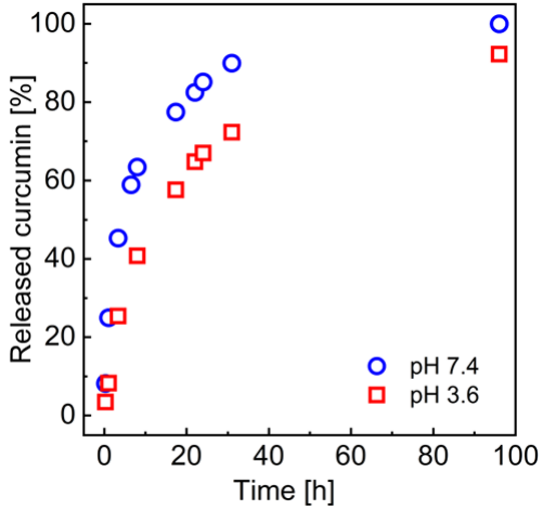
Cur release profile from PS–PAA NPs (*c*_PS–PAA_ = 0.075 mg/mL) at 25 °C in pH 7.4 and pH 3.6.

To determine the release profile of Cur from the PS–PAA NPs, we applied the previously described methodology.^40^ This procedure allowed us to avoid the problems resulting from the very low solubility of Cur in aqueous solutions (and its tendency to stay in the polymer phase) and its fast degradation in a neutral or alkaline environment. The OA phase was introduced on the top of the Cur-loaded NP dispersion. Cur solubilization in the OA phase was caused by disintegration of NPs in contact with the organic phase and mimicked the situation where the NPs deliver Cur to the cell. The OA phase was regularly tested using UV–vis spectrophotometry to record changes in the Cur concentration over time. The results, presented as a percentage of the cumulative release, are shown in Figure 5.

No “burst” release was observed. For the first 5 h, the profile followed zero-order kinetics (constant release rate), while for longer times, a non-linear release was observed. In the case of higher pH, Cur was completely released after 100 h. At the lower pH, we observed slower drug release, which may be related to the NPs’ aggregation.

### Distribution of Cur inside the PS Core

**Figure 6:**
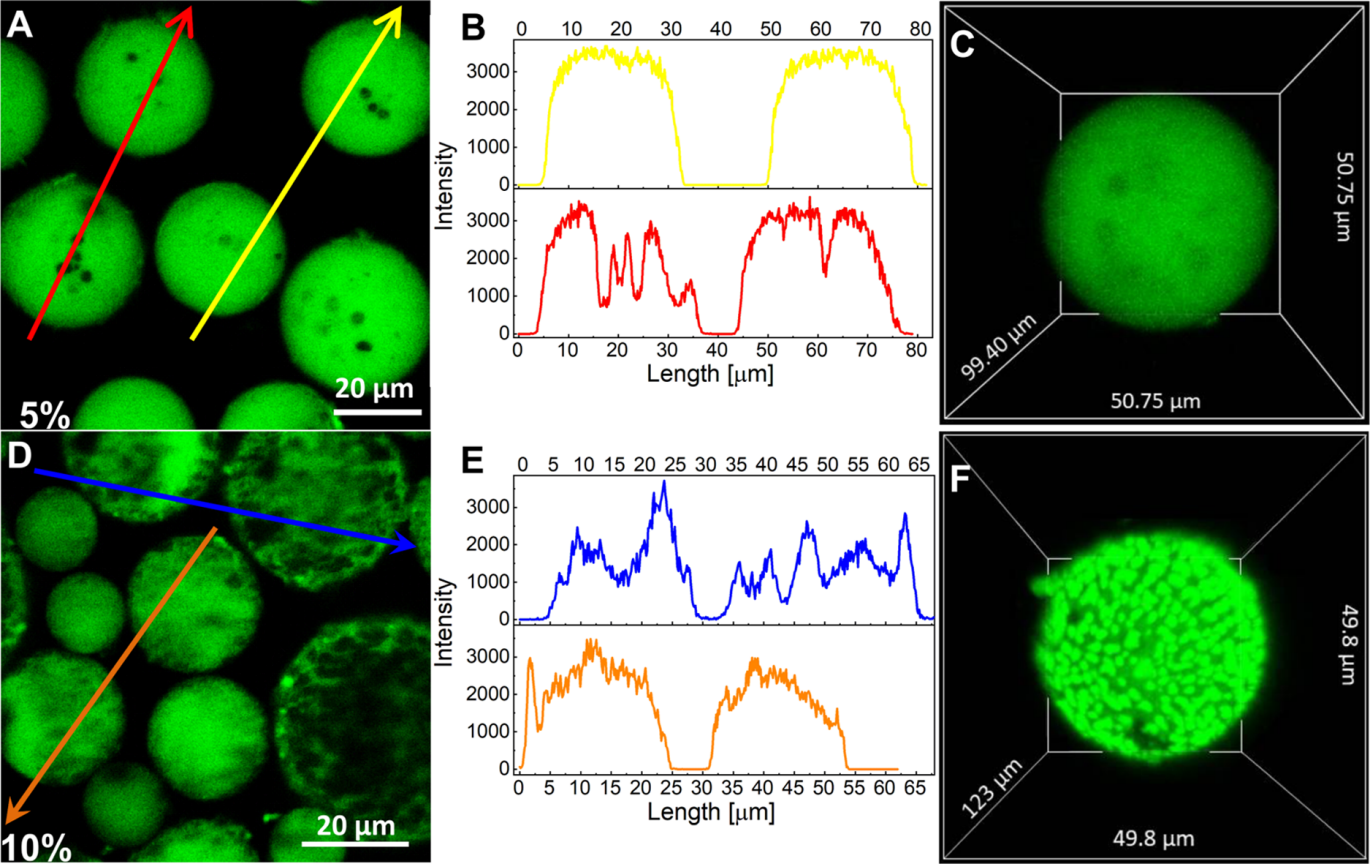
Confocal micrographs of Cur-loaded PS MPs formed in the presence of 5% (A) and 10% (D) Cur (Cur fluorescence is shown in green) and fluorescence intensity profiles (B,E) along the arrows shown in panels (A,D). Three-dimensional images of the Cur-loaded PS MPs formed in the presence of 5% (C) and 10% (F) Cur constructed from a series of confocal micrographs.

Cur exhibits relatively strong fluorescence in the visible region, so we used optical and laser scanning confocal microscopy (LSCM) to study the distribution of the drug in the PS matrix. The PS–PAA NPs are too small to be observed under an optical microscope; therefore, in this experiment, we used the MPs obtained from polystyrene as a model of the PS core. Figure S4 shows that the application of the emulsion solvent evaporation method from an aqueous dispersion containing PVA as an emulsion stabilizer produced spherical PS particles with diameters in the range of 63.7 ± 11.4 μm. Small pores are visible inside the PS MPs, formed most likely during dichloromethane evaporation, which is consistent with the previous research.^41^ To prepare Cur-loaded PS MPs, a constant PS concentration (1 mg/mL) and a variable Cur content (5, 10, and 30 wt % with respect to the weight of the polymer) were used. Figure 6 depicts typical micrographs of the Cur-loaded MPs. LSCM imaging shows large differences in the MP morphology and Cur distribution in the MPs depending on the amount of curcumin loaded. For the lowest Cur content (5 wt % in the feed), the MPs were mostly spherical with an average diameter of 30.6 ± 7.7 μm. The drug was evenly distributed within the whole volume of the structures, as indicated by the cross-sectional fluorescence intensity (Figure 6B). As Cur was only enclosed in the polymeric matrix, the small pores inside the PS MPs (resulting from the evaporation of dichloromethane) were drug free. Increasing the amount of entrapped Cur caused the PS MPs to be less spherical in shape, but their average size changed only slightly to 25.1 ± 8.8 μm. However, an uneven distribution of Cur in the MPs was observed. Cur accumulated in domains that are distributed throughout the volume of PS microstructures (Figure 6F). Introducing a much larger amount of the drug (30 wt % in the feed) caused much greater changes in the morphology of the structures (Figure S5). The observed objects were irregular in shape. The LSCM image shows that during the preparation procedure, Cur formed large aggregates that were enclosed in the polymer structures. Interestingly, the encapsulation efficiency (EE) values (Table 2) indicate that the EE of Cur increases significantly with the increasing amount of the drug used in the formulation. This is because the drug is retained in the MPs in the form of aggregates. The greater amount of Cur facilitates the formation of its aggregates inside the emulsion droplets.

In addition, we investigated the possibility of partitioning of Cur from the aqueous phase into the PS matrix. To this end, the empty PS MPs were treated with a Cur solution (*c*_Cur_ = 0.091 g/L) for 1 day. Figure S6 shows that the drug was mainly in the aqueous phase and the PS MPs did not fluoresce. Concluding, the entry of the drug into the already formed PS structures is strongly hindered.

### MD Simulations

#### Self-Organization of PS Oligomers in the Aqueous Phase

As Cur can accumulate only in the PS matrix, atomistic MD simulations of the system consisting of 14 PS oligomers placed in an aqueous phase were carried out to better understand the molecular organization of the hydrophobic core of the PS–PAA NPs. Figure S2 shows that the oligomers aggregated progressively during the simulation to form a single polymer coil. The time taken by the PS oligomers to fully aggregate was determined from the behavior of the combined radii of gyration of all the oligomers in the system (Figure S7). These curves clearly flatten at about 60 ns, indicating that the aggregate reached a stable configuration at that point. Figure S8 shows the aggregate internal structure, representing each oligomer chain in a different color. The visualization indicates that the PS aggregate has a form of a cluster created by individual oligomer coils stuck together into an irregular, boat-like shape.

**Figure 7:**
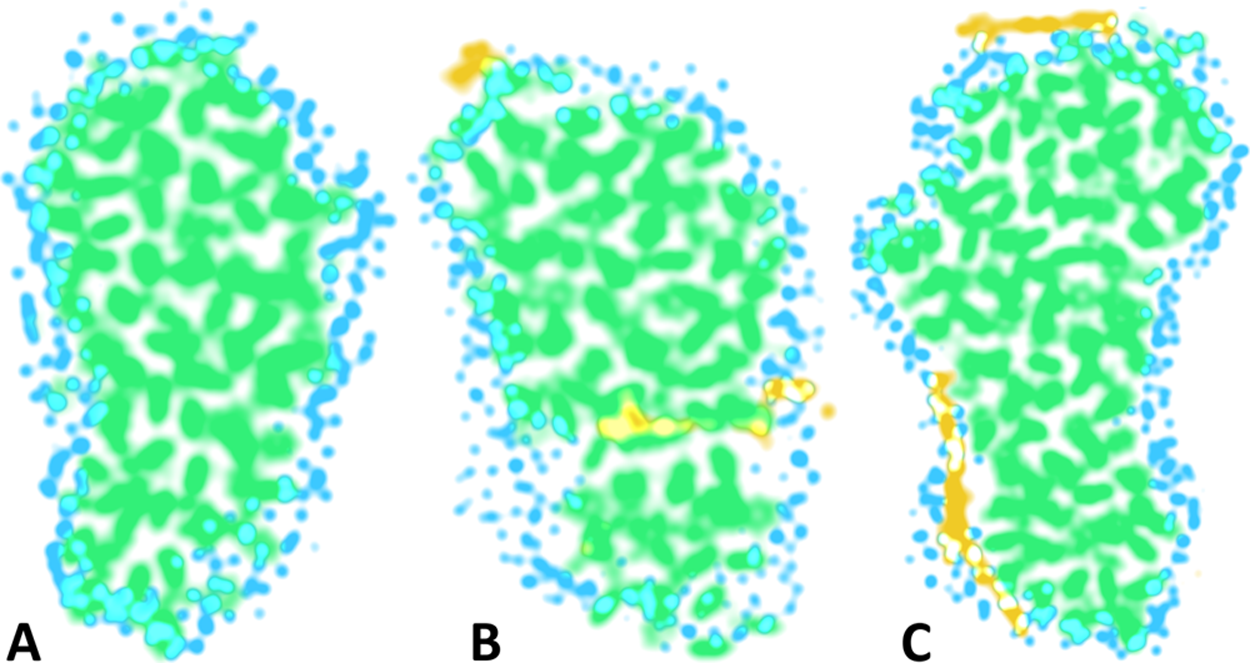
Two-dimensional maps of mass density of the PS aggregate in systems: PS_coil at *t* = 200 ns (A), Cur^0^–PS_disp at *t* = 500 ns (B), and Cur^0^–PS_coil at *t* = 200 ns (C). The maps show 0.8 nm-thick slices taken at the thickest point of the aggregate along the respective principal planes, with the density values rescaled for visual clarity. PS oligomers and Cur molecules are shown in green and yellow, respectively. Water molecules within the distance of 0.5 nm from the PS coil are shown in blue. The cyan areas represent overlapping of water and PS densities.

To gain insights into the internal structure of the aggregates, density maps in the three principal planes, that is, planes perpendicular to the principal axes, were calculated (Figure 7A). The maps reveal the presence of small voids inside the aggregate; however, they do not contain any water molecules. This confirms that the PS core is completely hydrophobic.

The irregular shape of the PS aggregate made it difficult to calculate its density, which is usually calculated in terms of the radial or cylindrical mass density distribution. For this reason, an indirect approach was implemented: the volume of the PS coil was calculated as the difference between the total volume of the simulation box and the volume outside of the PS aggregate (external volume). After removing the solvent molecules from the simulation box, the volume was calculated with the “freevolume” GROMACS tool configured to disregard the interior volume of the PS aggregate. For this purpose, large radius of the probe was chosen so that it would not fit into the voids present inside the aggregate, thus excluding them from the calculation. Volumes were calculated for a series of radii; then, the true external volume was calculated as a value extrapolated to the probe radius of 0 nm (Figure S9). The aggregate volume averaged over the last 50 ns was 94 nm^3^, and the mass density of the PS aggregate was calculated to be about 1.12 g/cm^3^. This value is close to the experimentally determined density of 1.040–1.065 g/cm^3^ for polystyrene beads.^42^ Therefore, the PS aggregate can be considered as a good model of the hydrophobic core of the PS–PAA NPs.

#### Self-Assembly of PS Oligomers in the Presence of Cur

A simulation of the systems containing 12 Cur molecules, or 12 Cur anions distributed between the initially dispersed 14 PS oligomers (the Cur^0^–PS_disp and Cur^–^–PS_disp systems) were performed to check the drug entrapment process in the PS structures during their preparation. Snapshots taken at the beginning and the end of the simulations are shown in Figures 1A and S10A, respectively.

All the Cur molecules interacted very quickly with the PS oligomers, which simultaneously accumulated together to form smaller clusters and ultimately a stable aggregate. This indicates the strong affinity of the bioactive to the PS matrix. The 200 ns period was sufficient for full aggregation. However, the aggregate formed during this timeframe was quite bulky and loosely packed (Figure 1B). For this reason, the simulation was extended to 500 ns. After this time, the aggregate became more compact, as shown in Figure 1C. The two-dimensional mass density map of the PS aggregate in the Cur^0^–PS_disp system shows that the Cur molecules are partially immersed in the aggregate between the PS coils and partially adsorbed at the aggregate surface.

**Figure 8:**
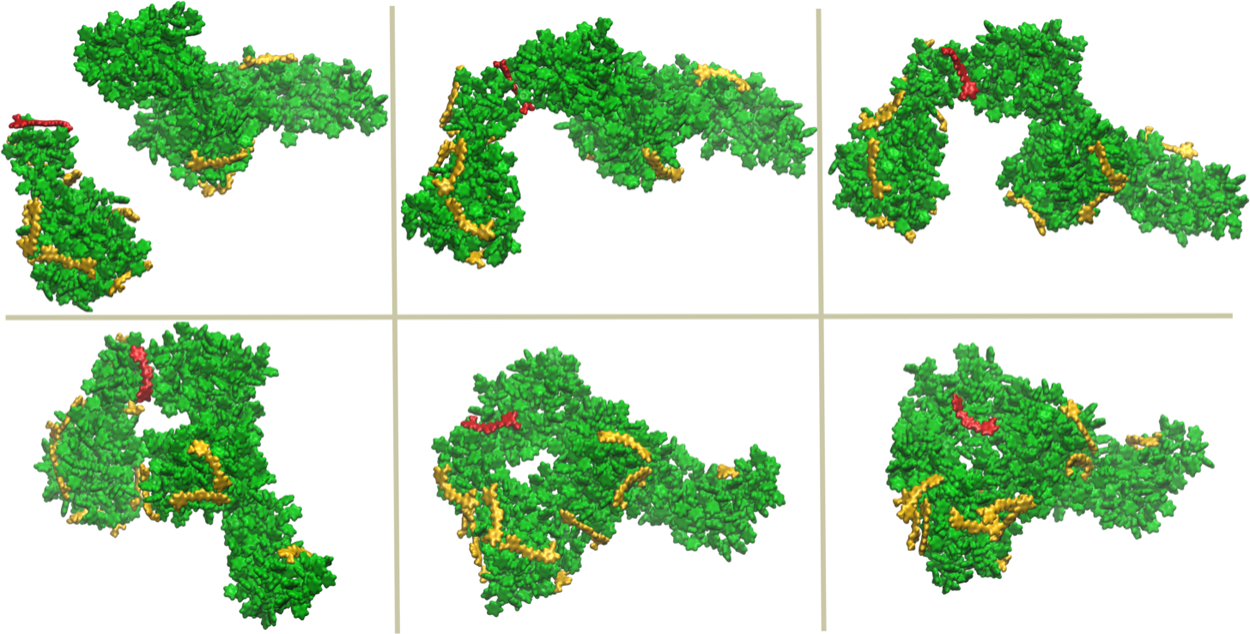
Docking event between two PS clusters showing an expulsion of an intercalated Cur molecule to the surface.

**Figure 9:**
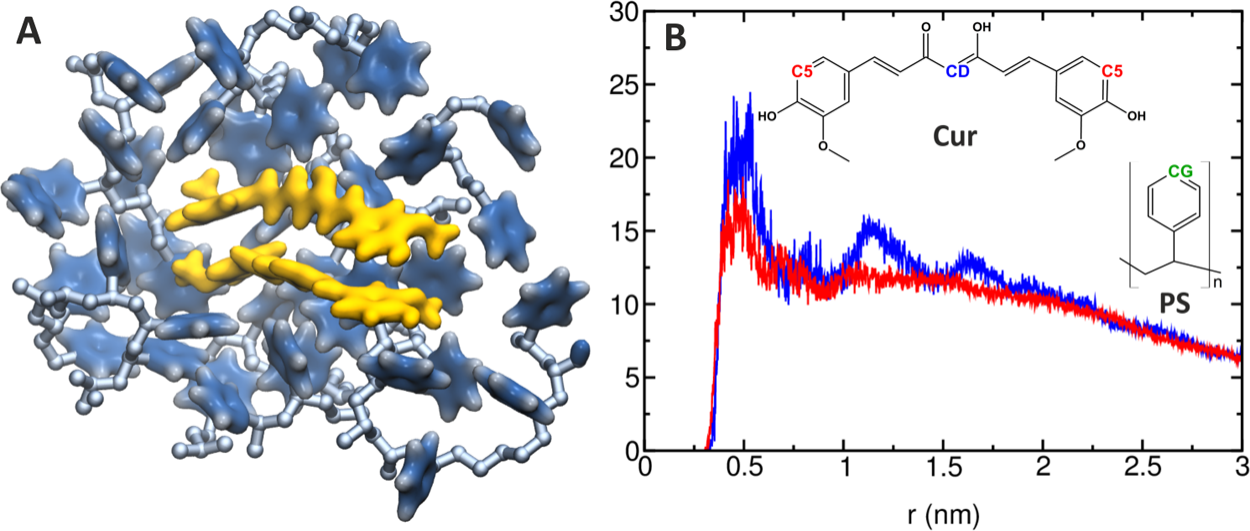
(A) Snapshot showing Cur dimer that formed during the simulations of the Cur^0^–PS_disp system. Cur is shown as a Quick Surface representation in yellow. PS phenyl groups in a distance of 1 nm from Cur are shown as a Quick Surface representation in blue. (B) Radial distribution function for the atomic pair CG–C5 (red line) and CG–CD (blue line) for one of the Cur^0^–PS_disp systems. The Cur and PS structures are shown as an inset.

A visual inspection of the trajectory showed that the merging of the smaller clusters was accompanied by a tendency to push the Cur molecules onto the polymer surface (Figure 8). It follows that the interactions between the PS moieties are apparently stronger than those that bind the Cur molecules to the PS matrix. This promotes gathering of drug molecules together and thus the formation of Cur dimers, possibly also larger aggregates. Cases of Cur aggregation inside the PS aggregate were observed in the MD simulations. Figure 9 shows the Cur dimer that formed inside the PS matrix in one of the Cur^0^–PS_disp systems. The observed dimers probably formed due to π-stacking interactions, as Cur phenyl rings are arranged nearly in parallel. In addition, the presence of drug molecules inside the PS aggregate forces the appropriate conformation of the polymer chains, in which the PS phenyl groups are directed toward the drug molecules (Figure 9). This confirms the earlier assumption that the enclosed drug changes the structure of the PS core, which enables drug accumulation. The aggregation of Cur observed in the MD simulations is also consistent with the microscopic observations, which showed that at higher drug concentrations, Cur is unevenly distributed inside the MPs.

To further analyze the Cur–PS interactions at the atomic scale, radial distribution functions (rdfs) were calculated for the two pairs of atoms (Figure 9B): the CG atom of the phenyl moieties in the PS molecule and two Cur atoms arbitrary selected to represent its phenyl rings (C5 atom) and the middle of the linking chain (CD atom) (see the inset in Figure 9B). Both rdfs show a maximum at 0.5 nm, which indicates the presence of a weak interaction between the drug and the PS phenyl groups. The average numbers of the polymer phenyl groups located within 0.7 nm from the Cur reference atoms are 3.0 and 3.4 for the C5 and CD atoms, respectively, indicating that both portions of the Cur molecules interact to a similar degree with the polymer phenyl rings.

#### Interaction of Cur with the PS Aggregate

We simulated the interactions of Cur^0^ and Cur^–^ with the already prepared PS aggregate to explain the possibility of post-incorporation of Cur into the PS matrix. Twelve drug molecules were irregularly placed around the PS aggregate (Figure 2A,C), and the systems were simulated for 200 ns. Figure 2B,D shows the snapshots taken at the end of the simulations. Both the neutral molecules and the anions, initially placed in the aqueous phase, migrated promptly to the interface of the PS aggregate and remained there for the rest of the simulation time. In the case of the neutral form, most of the Cur molecules adhered to the surface of the PS aggregate after approximately 50 ns, maintaining mostly the same distance from the center for the remainder of the simulation. The drug molecules did not display any tendency to move toward the center of the polymer aggregate.

**Figure 10:**
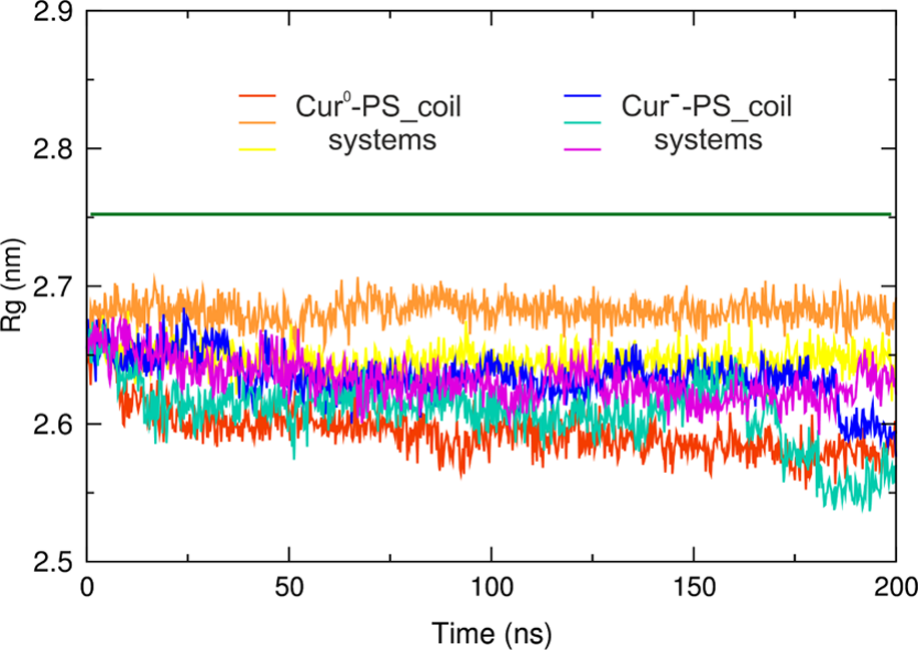
Total radius of gyration (*R*_g_) of the PS aggregate in the Cur^0^–PS_coil and Cur^–^–PS_coil systems. The green solid line marks the *R*_g_ of the empty PS aggregate averaged on the last 50 ns of the PS_coil system simulation.

The radius of gyration (*R*_g_) for the PS aggregate was calculated for all systems (Figure 10). In each system, the radius of gyration is between 2.55 and 2.70 nm, which is consistent with the measured distances of the Cur molecules with regard to the center of mass of the PS cluster. It should be noted, however, that due to the irregular shape of the aggregate, it is difficult to meaningfully measure the “radius from the center”. Importantly, the *R*_g_ values for Cur-containing aggregates are smaller compared to the empty PS aggregate. This indicates that the presence of Cur in the aggregate enforces its more compact structure.

### Cytotoxicity of PS–PAA NPs

**Figure 11:**
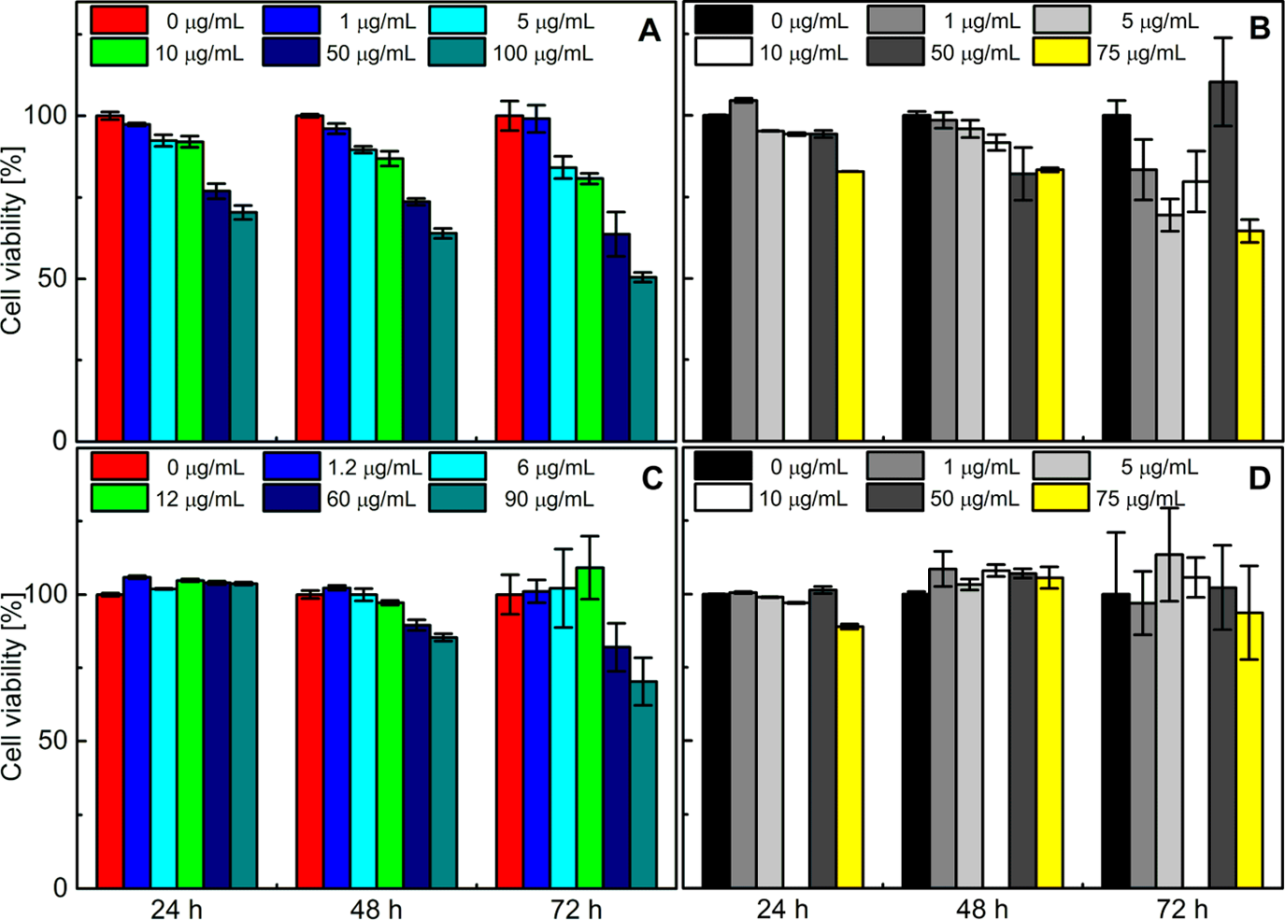
Cytotoxicity of the PS–PAA NPs (A,B) and Cur-loaded PS–PAA NPs (C,D) toward human skin fibroblasts (HSFs) (A,C) and A549 cells (B,D). Cells were incubated for 24, 48, and 72 h in medium containing various concentrations of the copolymer. Results are the mean ± SD of three experiments.

The cytotoxicity of polymers is one of the most important issues when considering them for biotechnological and biomedical applications.^43^ The empty and Cur-loaded PS–PAA NPs were tested for their effects on normal HSFs and cancer A549 cells. The MTT test, which allows assessing the metabolic activity of cells, was applied to examine the viability of cells incubated for 24, 48, and 72 h without (control) or with the NPs (Figure 11).

The empty PS–PAA NPs had only slightly negative influence on the HSF viability in the concentration range up to 10 μg/mL. For the higher concentrations, the cytotoxicity is more pronounced, although it is still very moderate after 48 h, while after 72 h, the viability of HSFs incubated with PS–PAA NPs in concentrations above 50 μg/mL was reduced by almost 50%. In the case of A549 cells, the 48 h incubation did not cause a significant decrease in cell viability in the polymer concentration range from 0 to 10 μg/mL. At higher concentrations, the A549 cell viability values were approximately 80% or higher. After 72 h of incubation, a visible decrease in cell viability was, however, observed for all concentrations tested.

Our results suggest a good biocompatibility of the PS–PAA copolymers, which is consistent with the studies on other cell lines. Previously, it was reported that the PS–PAA copolymer showed no cytotoxicity to the human colon cancer (Caco-2) cells, even in the sample whose concentration was higher than 5.0 mg/mL^11^ and to BeWo and bEnd3 cells.^9^

The viability of HSFs was not affected by incubation with the Cur-loaded PS–PAA NPs after 24 h (Figure 11C). Up to a concentration of 10 μg/mL, no cytotoxic effect was observed on HSFs for these NPs even after 72 h of incubation. The small drop in cell viability was observed for the two highest concentrations of PS–PAA (60 and 90 μg/mL) after 48 h, while after 72 h, this negative effect was even greater. Compared with the empty carrier, it can be concluded that loading of the PS–PAA NPs with Cur has a preventive effect on cell viability. When encapsulated in the NPs, Cur did not decrease the viability of the A549 cells (Figure 11D). On the contrary, its positive influence was noticed, with the most pronounced change observed after 72 h.

To conclude, the PS–PAA NPs show no significant cytotoxicity in the first 24 h, but a significant decrease in cell viability was observed after 48 h for all the concentrations studied. As expected, their toxicity was more pronounced in the normal cells in comparison to the cancer cell line. The encapsulated Cur has positive influence over the viability of the cells in contact with the PS–PAA NPs regardless of the cell type (HSF or A549).

## Conclusions

In this study, we characterized the loading capacity of PS–PAA NPs with hydrophobic drugs and distribution and possible aggregation of hydrophobic drug molecules inside the PS matrix. The microscopic visualization showed that the PS–PAA copolymer with *w*_hydrophilic_ ∼ 0.15 can self-assemble into NPs with a non-homogenous hydrophobic core of ∼35 nm in diameter surrounded by a hydrophilic PAA corona. The PS–PAA NPs are stable at physiological pH. The MD simulations showed that the hydrophobic core of PS–PAA NPs consists of individually coiled polymer chains that are adhered to each other. Such a compact structure of the hydrophobic core strongly hindered the post-incorporation of the drug to the already prepared PS–PAA NPs. However, both the neutral form of curcumin and its anion have good affinity for the PS matrix and the drug can be effectively enclosed in the PS–PAA NPs during their preparation. There are no specific interactions between the core-forming PS and Cur; therefore, the drug is incorporated into the PS matrix due to its hydrophobicity. The PS–PAA NPs can accumulate Cur with a reasonable DLC of ∼11 wt %. The DLC value is likely limited by the rigid structure of the PS core. The LSCM visualization showed that Cur is evenly distributed over the PS matrix only at low content (<2 wt %). Increasing the Cur concentration causes the formation of drug aggregates in PS–PAA structures, thus the non-uniform drug distribution in the PS matrix. We found PS–PAA to be relatively low cytotoxic to human skin fibroblasts. An interesting outcome of our studies is that the incorporation of Cur in the PS–PAA structures reduced their cytotoxicity. This is a good starting point for further research on the application of the Cur-loaded PS–PAA NPs for the delivery of selected cytostatic drugs; it is known that curcumin can augment the cytostatic, cytotoxic, and anti-invasive effects of cytostatics on drug-resistant cancer cells.^44^ In summary, the PS–PAA copolymer can be applied to prepare an effective Cur carrier for possible drug delivery applications.

## Experimental Section

### Materials

Polystyrene (PS, 35 kDa), poly(styrene)–*block***–**poly(acrylic acid) (PS-PAA) (*M*_w_ = 83 500, *M*_w_ of PS block = 70 500, *M*_w_ of PAA block = 13 000, and PDI ≤1.1), curcumin (Cur, curcuminoid content ≥ 94%, Cur content ≥ 80%), oleic acid (OA, 90%), Dulbecco’s modified Eagle’s medium (DMEM)—low glucose, MTT suitable for cell culture (≥97.5% HPLC), and PBS (tablets) were purchased from Sigma-Aldrich and used as received. Poly(vinyl alcohol) (PVA, *M*_w_ 14 000, 98.5–100% degree of hydrolysis) was obtained from BDH Chemicals. Sodium dodecyl sulfate (SDS, ≥ 99%) was purchased from BioSchop. Millipore-quality water was used in all experiments.

### Preparation of PS–PAA NPs

The PS–PAA copolymer (12.5 mg) was dissolved in tetrahydrofuran (THF, 1 mL). Deionized water (200 μL) was added dropwise to the stirred solution. The dispersion was continuously stirred for 2 h. Deionized water (4.8 mL) was then added. After stirring for 10 min, the sample was placed in a dialysis tube and dialyzed for 3 days against deionized water. In the case of Cur-loaded NPs, a solution of Cur in THF (1 or 3.75 mg/mL) was used to dissolve the copolymer.

### Preparation of PS MPs

PS (50 mg) was dissolved in dichloromethane (4 mL). This solution was added dropwise to a 1% PVA solution (50 mL) stirred with a magnetic stirrer (400 rpm). The mixture was left overnight to evaporate the organic solvent. The PS MPs were centrifuged at 10,000 rpm in three cycles (3 × 20 min). In the case of Cur-loaded MPs, an appropriate amount of Cur (5, 10, and 30% w/w) was dissolved with the polymer in dichloromethane. To test the possibility of including Cur into the already prepared PS MPs, a solution of Cur in DMF (1 mg/mL, 20 μL) was added to the NPs’ dispersion (200 μL) and incubated for 24 h.

### Determination of DLC and EE

The DLC and EE values were determined as previously described.^29^ Briefly, lyophilized micro- or nanostructures containing Cur were weighted into a vial, and DMF was added to a concentration of about 2.5 g/L. The sample was shaken for 30 min (350 min^–1^) and then centrifuged (5 min, 10,000 rpm). The Cur concentration in the supernatant was determined using a spectrophotometer. The DLC and EE parameters were calculated using the following equations
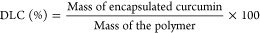
1
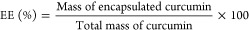
2

### Cryogenic Transmission Electron Microscopy

Cryogenic transmission electron microscopy (Cryo-TEM) was performed using a Tecnai F20× TWIN microscope (FEI Company, USA) equipped with a field emission gun operating at a 200 kV acceleration voltage. Images were recorded with an Eagle 4k HS camera (FEI Company, USA) and processed using the TIA software (FEI Company, USA). Prior to use, the grids with a holey carbon film (Quantifoil R 2/2; Quantifoil Micro Tools GmbH, Germany) were activated for 15 s in oxygen plasma using a Femto plasma cleaner (Diener Electronic, Germany). Samples were prepared by applying a droplet (3 μL) of the solution to the grid, blotting with filter paper, and rapid freezing in liquid ethane using a Vitrobot Mark IV (FEI Company, USA). After preparation, the vitrified specimens were kept under liquid nitrogen until they were inserted into a Gatan 626 cryo-TEM-holder (Gatan Inc., USA) and analyzed under the microscope at −178 °C.

### Dynamic Light Scattering

The light scattering analysis of NPs was performed with a Malvern Nano Zetasizer (Malvern Instruments Ltd.). Measurements were carried out in polystyrene cuvettes as described previously.^45^

### Optical and Laser Scanning Confocal Microscopy

The microscopic visualization of the samples was performed using a Nikon Eclipse T*i*-E inverted microscope coupled with an A1 scanning confocal system (Nikon, Japan) as previously described.^29^ A small drop of the sample was placed on the glass bottom dish (Nunc, Thermo Fisher).

### Release Studies

The dispersion of Cur-loaded NPs (6 mL) was placed in a glass vial and oleic acid (4 mL) was added. Due to the difference in the density of both phases, oleic acid constituted the upper layer of the system. The dispersion (the bottom phase) was constantly stirred using a magnetic stirrer. The oil phase (1.5 mL) was taken at regular intervals (15 min–96 h), its absorbance was measured using a Varian Cary 50 spectrophotometer, and the sample was gently returned to the cylinder, so as not to disturb the two-phase system. The calibration curve (*R*^2^ = 0.995, concentration range 0.8–6 μg/mL) was used to determine the Cur concentration in the oleic phase.

### MTT Assay

Human skin fibroblasts (HSFs, ATCC CRL-2522) and adenocarcinomic human alveolar basal epithelial cells (A549, ATCC CCL-185) were cultured in DMEM (Sigma-Aldrich) in a humidified incubator under standard conditions (37 °C and 5% CO_2_) in all in vitro experiments. The medium was supplemented with 10% fetal bovine serum (FBS; Gibco; Thermo Fisher Scientific) and 0.1% penicillin/streptomycin cocktail (Sigma-Aldrich). The cells were sub-cultured every 2 days until the appropriate number of cells for testing was obtained. The cells were then trypsinized (0.25% trypsin with ethylenediaminetetraacetic acid—EDTA, Corning), centrifuged (300 g, 5 min), suspended in fresh medium, seeded on sterile 96-well plates (1.5 × 10^4^ cells/cm^2^), and incubated for next 24 h. After incubation, the medium was replaced with fresh DMEM containing varying concentrations of empty or Cur-loaded NPs. The cells incubated in standard medium were used as control. After 24, 48, and 72 h of incubation, cell viability was measured using the MTT assay (according to the manufacturer’s protocol). A sterile solution of 3-(4,5-dimethylthiaziazol-2-yl)-2,5-diphenyl tetrazolium bromide (5 mg/mL) was mixed with the medium at a 1:10 (v/v) ratio and used to replace the medium with NPs. After 4 h of incubation in 37 °C (5% CO_2_), a 10% solution (w/v) of sodium dodecyl sulfate was added to dissolve formazan crystals. After another 4 h, the absorbance of each well containing dissolved formazan crystals was measured at 570 nm using a microplate reader Multiskan FC (Thermo Fisher Scientific).

## Supporting Information Available

Structure of the PS residues, snapshots of the PS_coil systems, emission spectra of Cur in PBS and in PS–PAA NPs, optical microscopy image of PS MPs, optical and confocal micrographs of the Cur-loaded PS MPs, confocal micrographs of the PS MPs treated with the Cur solution, polystyrene radii of gyration in the PS_coil system, PS aggregate structure formed in the PS system, PS aggregate external volume plot as a percentage of total box volume, snapshots of the Cur^–^–PS_disp system, and atom types and charges for styrene (PDF).
