# Supplementary material for: Encapsulation of Curcumin in Polystyrene-Based Nanoparticles—Drug Loading Capacity and Cytotoxicity
Source: ACS Omega. 2021 Apr 29;6(18):12168–78. doi: 10.1021/acsomega.1c00867 (PMC8154162; doi:10.1021/acsomega.1c00867)
Supplement: Supplementary file 1 — ao1c00867_si_001.pdf [file ao1c00867_si_001.pdf]

Supporting Information

# Encapsulation of Curcumin in Polystyrene-Based Nanoparticles – Drug Loading Capacity and Cytotoxicity

*Maria Zatorska-Płachta,[†] Grzegorz Łazarski,[†] Urszula Maziarz,[†] Aleksander Foryś,[‡] Barbara Trzebicka,[‡] Dawid Wnuk,[⊥] Karolina Chołuj,[†] Anna Karewicz, [†] Marta Michalik,[⊥] Dorota Jamróz,[*,†] and Mariusz Kepczynski[*,†]*

[†] Jagiellonian University, Faculty of Chemistry, Gronostajowa 2, 30-387 Kraków, Poland

[‡] Centre of Polymer and Carbon Materials, Polish Academy of Sciences, M. Curie-Sklodowskiej 34, 41-819 Zabrze, Poland

[⊥]Department of Cell Biology, Faculty of Biochemistry, Biophysics and Biotechnology, Jagiellonian University, Gronostajowa 7, 30-387 Kraków, Poland

The CHARMM forcefield includes parameters for styrene. Since styrene contains a stereogenic carbon atom, two residues, STYRA and STYRB were created to replicate this effect. Their parameters are identical, except for slight differences in both geometrical and topological atom arrangement required to capture the tacticity of the oligomer chain.

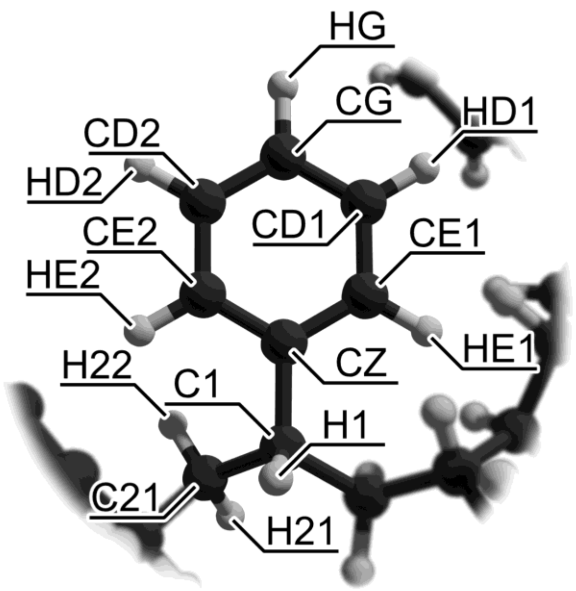


**Figure S1**. Visualization of the structure of the PS residues, with listed atom types.

**Table S1**. Atom types and charges for PS Styrene (STYRA & STYRB)

| Element | Symbol | Type | Charge |
|---|---|---|---|
| C | C2 | CG321 | -0.180 |
| H | H21 | HGA2 | 0.090 |
| H | H22 | HGA2 | 0.090 |
| C | C1 | CG311 | -0.090 |
| H | H1 | HGA1 | 0.090 |
| C | CZ | CG2R61 | 0.000 |
| C | CE1 | CG2R61 | -0.115 |
| C | CD1 | CG2R61 | -0.115 |
| C | CG | CG2R61 | -0.115 |
| C | CD2 | CG2R61 | -0.115 |
| C | CE2 | CG2R61 | -0.115 |
| H | HE1 | HGR61 | 0.115 |
| H | HD1 | HGR61 | 0.115 |
| H | HG | HGR61 | 0.115 |
| H | HD2 | HGR61 | 0.115 |
| H | HE2 | HGR61 | 0.115 |
| | $CH_3$ terminal | | |
| C | C2 | CG331 | -0.270 |
| H | H21 | HGA3 | 0.090 |
| H | H22 | HGA3 | 0.090 |
| | H terminal | | |
| C | C1 | CG321 | -0.180 |
| H | H1 | HGA2 | 0.090 |

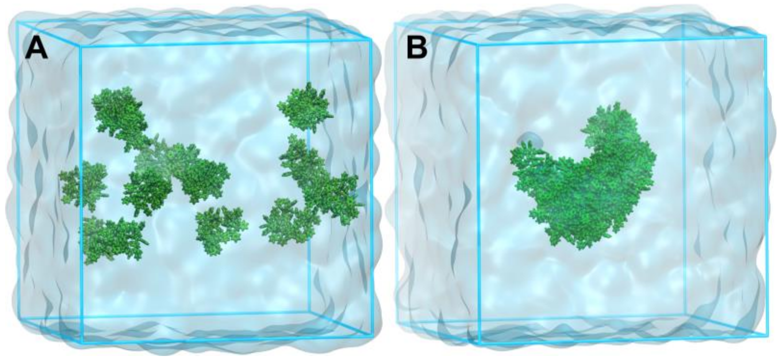


**Figure S2.** Snapshots of initial (A) and final (B) configurations of the PS_coil systems. Oligomers are shown in green. For clarity, water molecules are shown as a continuous blue phase.

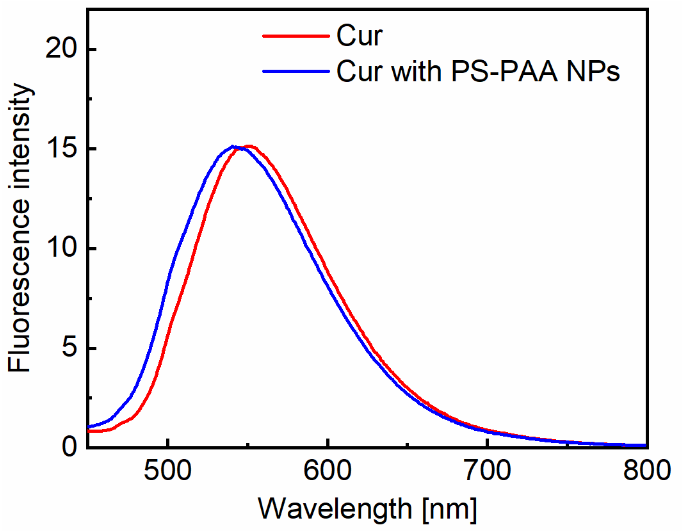


**Figure S3**. Emission spectra of Cur ($c_{Cur}$ = 13.6 µM, $\lambda_{exc}$ = 429 nm) in PBS before (red line) and after incubation with the PS-PAA NPs ($c_{PS\text{-}PAA}$ = 0.09 mg/mL, blue line).

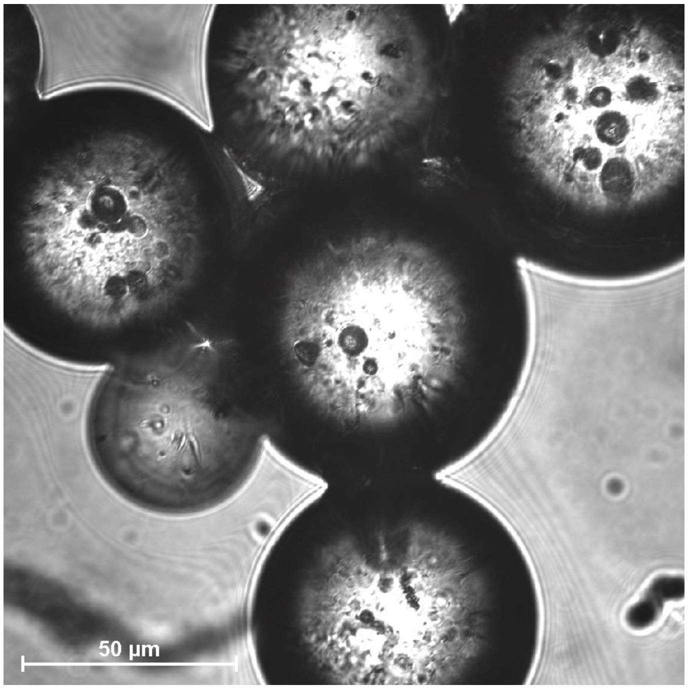


**Figure S4**. Optical microscope image of PS microparticles prepared by the emulsion solvent evaporation method from an aqueous dispersion containing PVA as an emulsion stabilizer. Small pores inside the microparticles are visible.

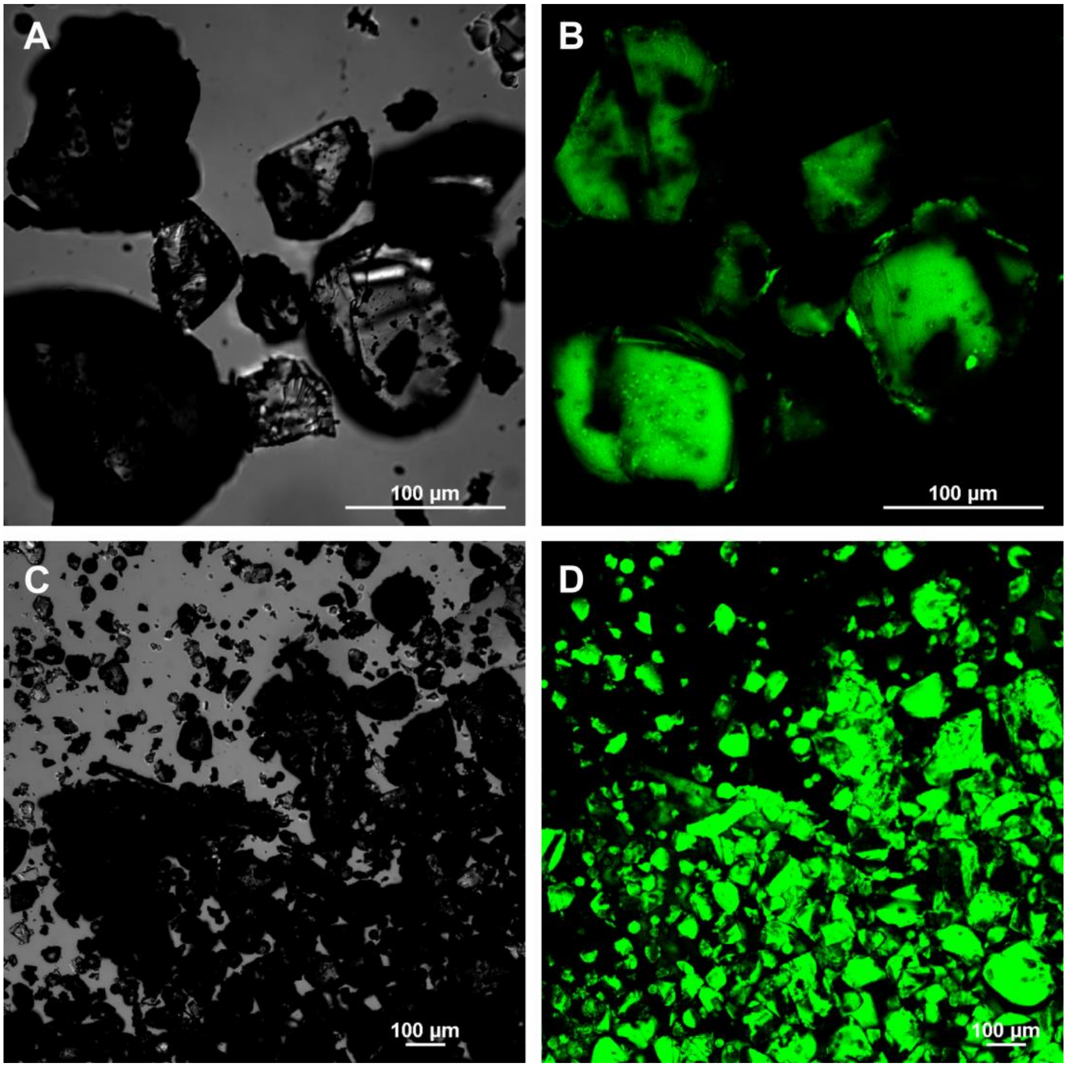


**Figure S5**. Optical (A, C) and confocal (B, D) micrographs of the Cur-loaded PS microparticles prepared in the presence of 30 wt-% Cur. Fluorescence of Cur is shown in green.

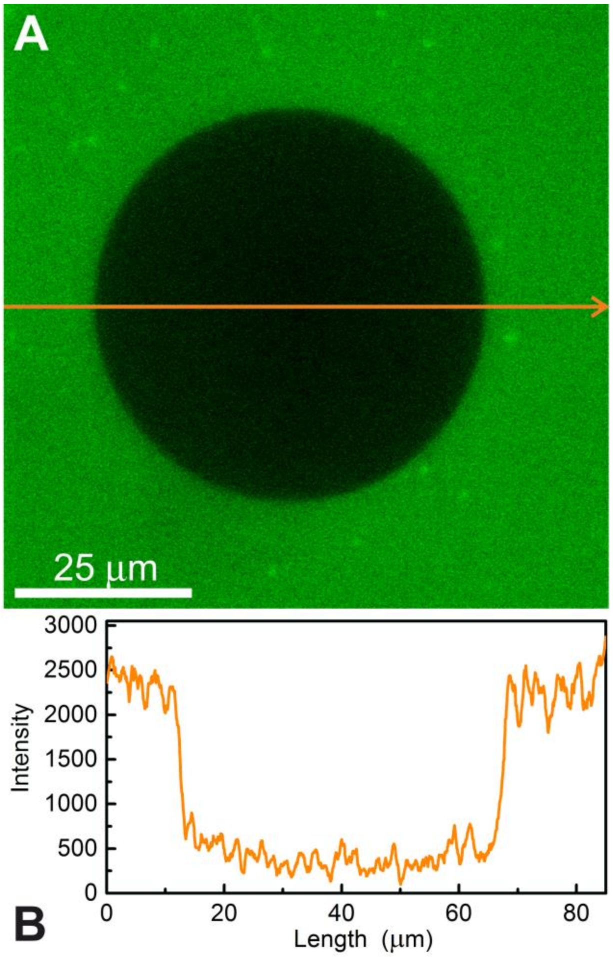


**Figure S6**. (A) Confocal micrographs of the PS microparticles treated with the Cur solution in PBS/DMF 10:1 ($c_{Cur}$ = 0.091 g/L) for one day and (B) fluorescence intensity profile along the arrow shown in panel A. Fluorescence of Cur is shown in green.

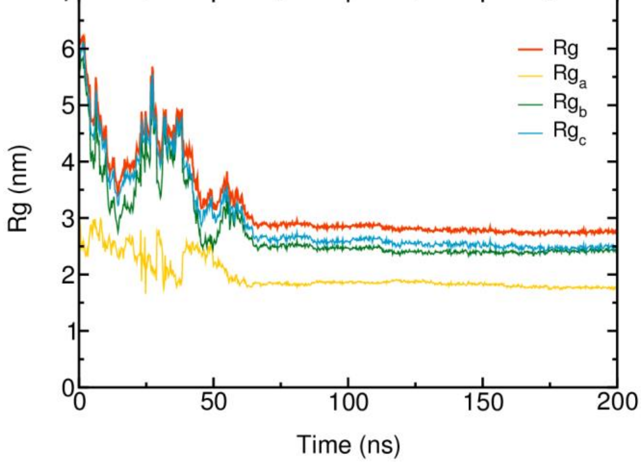


**Figure S7.** Polystyrene radii of gyration in the PS_coil system as a function of time: total ($R_g$) and the radii around the principal axes ($Rg_a$, $Rg_b$ and $Rg_c$).

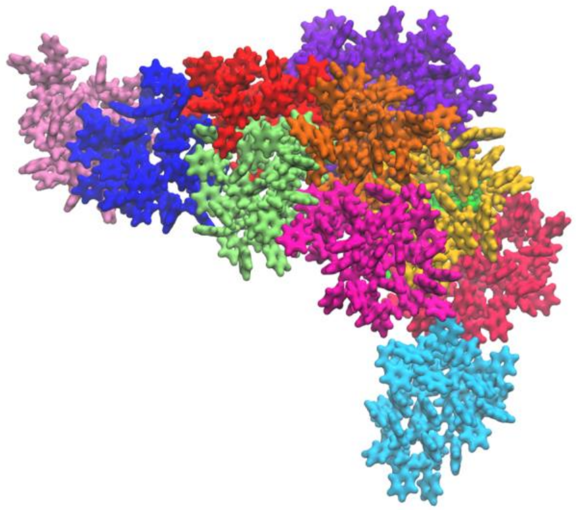

**Figure S8.** Structure of the PS aggregate formed in the **PS** system after 200 ns of simulation. Each individual PS oligomer is shown in a different color. Water is not shown for clarity.

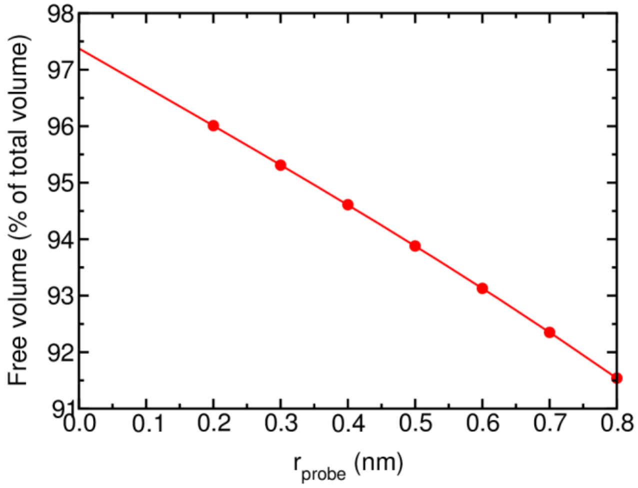


**Figure S9.** PS aggregate external volume plot as a percentage of total box volume. The “true” external volume was obtained by extrapolating the probe radius to 0.

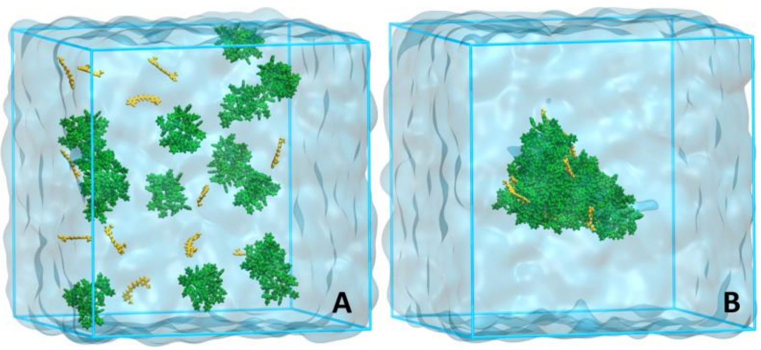


**Figure S10**. Snapshots of the configurations of the Cur⁻-PS_disp system at $t = 0$ (A) and 200 ns (B). The Cur molecules and PS oligomers are shown in yellow and green, respectively.
